# (Magneto)Transport Properties of (TiZrNbNi)_1−*x*_Cu_*x*_ and (TiZrNbCu)_1−*x*_Co_*x*_ Complex Amorphous Alloys

**DOI:** 10.3390/ma16041711

**Published:** 2023-02-18

**Authors:** Marko Kuveždić, Emil Tafra, Ignacio A. Figueroa, Mario Basletić

**Affiliations:** 1 Department of Physics, Faculty of Science, University of Zagreb, Bijenička Cesta 32, 10000 Zagreb, Croatia; 2 Instituto de Investigaciones en Materiales, Universidad Nacional Autónoma de México (UNAM), Circuito Exterior s/n, Cd. Universitaria, Ciudad de México 04510, Mexico

**Keywords:** compositionally complex alloys, amorphous alloys, metallic glasses, hall effect, superconductivity, electronic properties, variable range hopping

## Abstract

We present a systematic study of electrical resistivity, superconductive transitions and the Hall effect for three systems of compositionally complex amorphous alloys of early (TE) and late (TL) transition metals: ${\left(\mathrm{TiZrNbNi}\right)}_{1-x}{\mathrm{Cu}}_{x}$ and ${\left(\mathrm{TiZrNbCu}\right)}_{1-x}{\mathrm{Co}}_{x}$ in a broad composition range of 0<x<0.5 as well as ${\mathrm{Ti}}_{0.30}{\mathrm{Zr}}_{0.15}{\mathrm{Nb}}_{0.15}{\mathrm{Cu}}_{0.2}{\mathrm{Ni}}_{0.2}$, ${\mathrm{Ti}}_{0.15}{\mathrm{Zr}}_{0.30}{\mathrm{Nb}}_{0.15}{\mathrm{Cu}}_{0.2}{\mathrm{Ni}}_{0.2}$ and ${\mathrm{Ti}}_{0.15}{\mathrm{Zr}}_{0.15}{\mathrm{Nb}}_{0.30}{\mathrm{Cu}}_{0.2}{\mathrm{Ni}}_{0.2}$. All samples showed high resistivity at room temperature, 140–240 μΩ cm, and the superconducting transition temperatures decreased with increasing late transition metal content, similar to binary amorphous and crystalline high-entropy TE-TL alloys. The Hall coefficient $R_{H}$ was temperature-independent and positive for all samples (except for (TiZrNbCu)${}_{0.57}$Co${}_{0.43}$), in good agreement with binary TE-TL alloys. Finally, for the temperature dependence of resistivity, as far as the authors are aware, we present a new model with two conduction channels, one of them being variable range hopping, such as the parallel conduction mode in the temperature range 20–200 K, with the exponent p=1/2. We examine this in the context of variable range hopping in granular metals.

## 1. Introduction

Over the decades, metallic glass alloys of early (TE) and late (TL) transition elements have been extensively studied [1,2,3], and the interest further increased after the discovery of TE-TL-base bulk metallic glasses [4]. These alloys are distinguished by a broad range of compositions (typically 20–70% of the TL content) that form metallic glasses [5], which permits detailed systematic studies of the composition dependence of the electronic structure and mechanical and functional properties.

More recently, the development of new materials has shifted from traditional binary and ternary alloys to multicomponent compositionally complex alloys. First employed in an effort to discover new bulk metallic glasses [6,7], this shift soon expanded to crystalline high-entropy alloys [8,9] and eventually to intermetallic compounds and ceramics [10,11]. These new multicomponent metallic glasses offer interesting new research prospects. They allow us to expand our understanding of the effects of composition changes and chemical complexity on material properties, and they open up the possibility of comparisons with high-entropy alloys, which posses chemical but not structural disorder [12,13,14].

Furthermore, their excellent mechanical properties and phase stability in a large temperature range makes them interesting for technological application, such as magneto-caloric devices [15] and antidiffusion and anticorosive coatings (see, for example, [16] for a review). Continuing our work on multicomponent compositionally complex metal alloys [17,18,19,20,21,22], we report here on the results of a systematic study of electronic properties (the electrical resistivity, superconductive transitions and Hall effect) on amorphous samples of two quinary TE-TL alloy systems ${\left(\mathrm{TiZrNbNi}\right)}_{1-x}{\mathrm{Cu}}_{x}$ and ${\left(\mathrm{TiZrNbCu}\right)}_{1-x}{\mathrm{Co}}_{x}$ (from hereon ${\mathrm{Cu}}_{x}$ and ${\mathrm{Co}}_{x}$, respectively).

Additionally, we report on a set of thin ribbons prepared with the same procedures with nominal compositions: ${\mathrm{Ti}}_{0.30}{\mathrm{Zr}}_{0.15}{\mathrm{Nb}}_{0.15}{\mathrm{Cu}}_{0.2}{\mathrm{Ni}}_{0.2}$, ${\mathrm{Ti}}_{0.15}{\mathrm{Zr}}_{0.30}{\mathrm{Nb}}_{0.15}{\mathrm{Cu}}_{0.2}{\mathrm{Ni}}_{0.2}$ and ${\mathrm{Ti}}_{0.15}{\mathrm{Zr}}_{0.15}{\mathrm{Nb}}_{0.30}{\mathrm{Cu}}_{0.2}{\mathrm{Ni}}_{0.2}$ (from hereon ${\mathrm{Ti}}_{0.30}$, ${\mathrm{Zr}}_{0.30}$ and ${\mathrm{Nb}}_{0.30}$, respectively). These alloys possess varying concentrations of TE elements while maintaining the same ratio of TE to TL as ${\left(\mathrm{TiZrNb}\right)}_{0.6}{\left(\mathrm{NiCu}\right)}_{0.4}$ (the same composition as ${\mathrm{Cu}}_{0.20}$ and ${\mathrm{Ni}}_{0.20}$). We compare our results to similar binary TE-TL metallic glass alloys.

Lastly, we propose an explanation of the negative temperature coefficient of the temperature dependence of the resistivity, a long-standing issue for an entire class of strong scattering amorphous alloys, which we feel is still not solved in a satisfactory way. Our proposition of the observed temperature dependence of resistivity is based on two parallel conducting channels, one being metallic-like and the other similar to variable range hopping (VRH). We show that this surprising appearance of VRH is reminiscent of the VRH in granular metals.

## 2. Experimental Details

Ingots of ${\mathrm{Cu}}_{x}$ and ${\mathrm{Co}}_{x}$ alloys were prepared from high-purity components (≥99.8 at.%) by arc melting in high-purity argon in the presence of a titanium getter. From these ingots, thin ribbons of seven different compositions of ${\mathrm{Cu}}_{x}$ (x=0.0, 0.12, 0.20, 0.25, 0.32, 0.43 and 0.50) and five of ${\mathrm{Co}}_{x}$ (x=0.10, 0.20, 0.25, 0.32 and 0.43) were fabricated by melt-spinning molten alloys on the surface of a copper roller in a pure He atmosphere. A detailed description of the preparation and results of structural (X-ray powder diffraction—XRD), chemical (scanning electron microscopy with energy dispersive spectroscopy—SEM/EDS) and thermal (differential scanning calorimetry—DSC) characterisation of these ribbons was reported previously [20,22]. XRD and DSC results revealed that all samples except for ${\mathrm{Cu}}_{0.0}$ were fully amorphous. The SEM/EDS images confirmed a random distribution of constituent elements down to a micrometer scale, and the calculated average concentrations were within 1 at.% of nominal.

This paper describes the structural, chemical and thermal properties; susceptibility; microhardness; and ultraviolet photoemission spectroscopy (UPS) of ${\mathrm{Ti}}_{0.30}$, ${\mathrm{Zr}}_{0.30}$ and ${\mathrm{Nb}}_{0.30}$ [23]. From these measurements, ${\mathrm{Ti}}_{0.30}$ and ${\mathrm{Zr}}_{0.30}$ appear fully amorphous, while a study of the structure factors using high-energy XRD (HEXRD) revealed a minor nanocrystalline phase in the amorphous matrix of ${\mathrm{Nb}}_{0.30}$.

Ribbon samples that were typically 6–8 mm long, 1–2 mm wide and ∼30 μm thick were mounted on a sample holder with “GE” varnish. Prior to mounting, the samples were thoroughly cleaned in an ultrasonic bath with acetone and isopropyl alcohol. The current and voltage wires were glued with silver paste onto the samples. The silver paste was allowed to dry at room temperature resulting in contact resistances up to 100 Ω. The voltage noise due to contact resistance was negligible compared to the full voltage across the sample in the case of resistance measurements but presented a significant component of small Hall voltage. Uncertainty due to this noise was incorporated in the error values of the Hall coefficient.

The thickness of the ribbon samples was determined from the sample mass, broad surface area and density (determined form the rule of mixtures [17]). Uncertainty due to the finite width of silver paste contacts and the error in determining the sample dimensions (width and thickness) were also considered when calculating the errors of absolute resistivity and the Hall coefficient as well as values derived from those.

The resistance and Hall effect measurements were performed in the variable temperature insert of a 16/18 T Oxford superconducting magnet system in the 1.3–300 K temperature range and in magnetic fields up to ±16 T. Low-temperature measurements down to 300 mK were performed in a ${\mathrm{He}}^{3}$ insert (Heliox, Oxford Instruments, Abingdon, United Kingdom) of the same magnet system. The temperature was measured by a LakeShore 340 Temperature Controller with a calibrated Cernox thermometer situated close to the samples.

Low-temperature superconducting transition measurements were performed with the low frequency (22 Hz) AC method with rms currents in the 20–200 μA range, while the resistance and Hall effect measurements, depending on the sample, were performed with either the low frequency AC or DC method with currents in the 200 μA–2 mA range. For both measurement methods, AC and DC, the current source was Keithley 6221, while the voltages were measured using dual-phase Signal Recovery 5210 and 7225 Lock-in amplifiers and Keithley 2182A nanovolmeters for the AC and DC methods, respectively. The magnetic filed was oriented perpendicular to the broad surface of the ribbon samples and to the current direction.

## 3. Results and Discussion

### 3.1. Room Temperature Resistivity and Hall Effect

**Figure 1:**
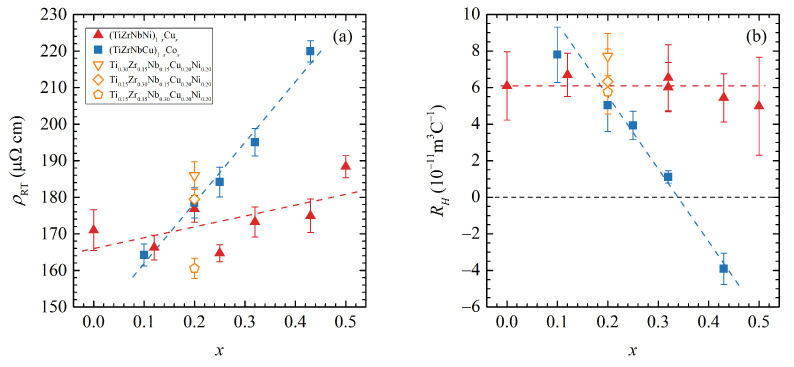
The composition *x* dependence of the room temperature resistivity $\rho_{\mathrm{RT}}$ (**a**) and Hall constant $R_{H}$ (**b**) for ${\left(\mathrm{TiZrNbNi}\right)}_{1-x}{\mathrm{Cu}}_{x}$ (red triangles), ${\left(\mathrm{TiZrNbCu}\right)}_{1-x}{\mathrm{Co}}_{x}$ (blue squares) and three additional alloys with fixed (CuNi) content (open triangles, diamonds and pentagons). The dashed lines are guides to the eye.

Figure 1a shows the room temperature resistivities of all studied amorphous alloys and their dependence on composition. All measured samples showed a high resistivity at room temperature (140–240 μΩ cm), as is usual for amorphous TE-TL alloys [24,25,26,27,28,29,30,31,32], and a small negative temperature coefficient of resistivity in agreement with the Mooij correlation [32,33]. The room temperature resistivities $\rho_{\mathrm{RT}}$ exhibited a certain amount of scatter between different samples of nominally the same composition with the error typically no more than around 5%.

For ${\mathrm{Cu}}_{x}$ alloys, the room temperature resistivities showed a tendency to increase slightly with increasing Cu content with both values and behaviour typical of binary TE-TL alloys [30,31] and similar to the ${\left(\mathrm{TiZrNbCu}\right)}_{1-x}{\mathrm{Ni}}_{x}$ [21] alloys.

On the other hand, for ${\mathrm{Co}}_{x}$ alloys, the resistivity increased significantly with increasing Co content. This is distinct from binary ${\mathrm{Zr}}_{1-x}{\mathrm{Co}}_{x}$ alloys which showed a smaller increase from 162 μΩ cm for x=0.20 to 183.5 μΩ cm for x=0.45 [27], and the difference is likely connected to the presence of Ti, Nb and Cu in our alloys. While it has been suggested [30] that increased s−d scattering is responsible for the increase in resistivity as one approaches the middle of the ${\mathrm{TE}}_{1-x}{\mathrm{TL}}_{x}$ phase diagram, it is unclear what roles the chemical composition and complexity have on the values of resistivity and the rate of increase with increasing TL content. Further study of more alloys (notably, ${\mathrm{Ti}}_{1-x}{\mathrm{Co}}_{x}$ and ${\mathrm{Nb}}_{1-x}{\mathrm{Co}}_{x}$) is needed to build a better understanding of the above-mentioned differences.

The last three alloys studied, with fixed (CuNi) content, showed a small resistivity decrease when going from ${\mathrm{Ti}}_{0.30}$ to ${\mathrm{Zr}}_{0.30}$ and then to ${\mathrm{Nb}}_{0.30}$, which is consistent with the resistivity changes found between binary alloys ${\mathrm{Ti}}_{1-x}{\mathrm{Ni}}_{x}$, ${\mathrm{Ti}}_{1-x}{\mathrm{Cu}}_{x}$, ${\mathrm{Zr}}_{1-x}{\mathrm{Ni}}_{x}$ and ${\mathrm{Zr}}_{1-x}{\mathrm{Cu}}_{x}$ and ${\mathrm{Nb}}_{1-x}{\mathrm{Ni}}_{x}$ (see [29,30,31] for data on some of those compounds and [32] for a general review on a number of various alloys).

The Hall voltage of all measured samples was linear with the magnetic field and essentially temperature-independent down to the lowest measured temperatures. The calculated values of Hall coefficient $R_{H}$ were positive—except for ${\mathrm{Co}}_{0.43}$. The Hall coefficient dependence on composition is shown in Figure 1b. $R_{H}$ drops rapidly for ${\mathrm{Co}}_{x}$ alloys with increasing Co content with an interpolated critical concentration (the concentration where $R_{H}$ changes sign) at $x_{c}=0.34$. In contrast, ${\mathrm{Cu}}_{x}$ alloys are generally constant in the measured range of the Cu concentration. Both results are in good agreement with binary TE-TL alloys: the ${\mathrm{Zr}}_{1-x}{\mathrm{Co}}_{x}$ exhibit a sharp drop with increasing Co content and a critical concentration of $x_{c}=0.32$ [34,35], and $R_{H}$ of ${\mathrm{Zr}}_{1-x}{\mathrm{Cu}}_{x}$, ${\mathrm{Ti}}_{1-x}{\mathrm{Cu}}_{x}$. ${\mathrm{Hf}}_{1-x}{\mathrm{Cu}}_{x}$ amorphous alloys stays mostly constant up to 50% Cu content with a critical concentration around $x_{c}=0.8$ [31,35,36].

As for the alloys with a fixed (CuNi) content, they are similar to the binary TE-Cu alloys where the $R_{H}$ also decreases when going from Ti to Zr and Nb [30,31]. The large change in $R_{H}$ values in ${\mathrm{Co}}_{x}$ alloys is consistent with a strong increase and a shift towards the Fermi level of the peak corresponding to the 3D state of Co in the UPS spectra [22]. On the other hand, the peak associated with the 3D states of Cu, which sits further from the Fermi level, shifts only slightly with increasing Cu content [20], which we associate with the nearly constant $R_{H}$ values in ${\mathrm{Cu}}_{x}$ alloys.

### 3.2. Superconductivity

**Figure 2:**
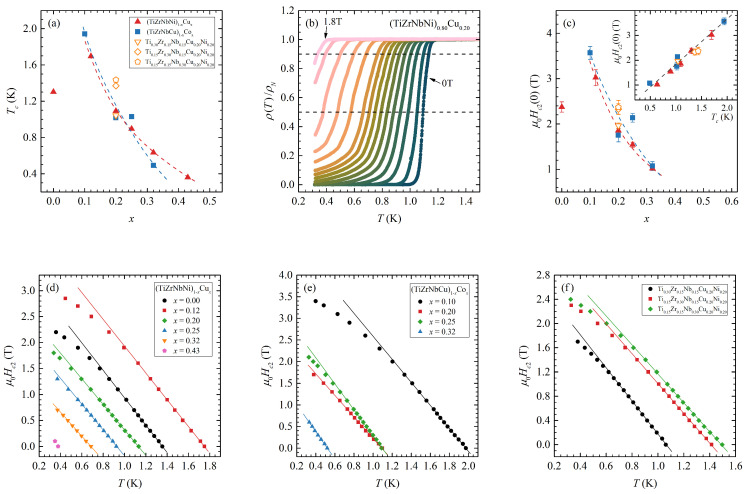
(**a**) The composition *x* dependence of the superconducting transition temperatures $T_{c}$ for ${\left(\mathrm{TiZrNbNi}\right)}_{1-x}{\mathrm{Cu}}_{x}$ (red triangles), ${\left(\mathrm{TiZrNbCu}\right)}_{1-x}{\mathrm{Co}}_{x}$ (blue squares) and three additional alloys with fixed (CuNi) content (open triangles, diamonds and pentagons). The dashed lines are guides to the eye. (**b**) The temperature dependence of the normalized resistivity $\rho \left(T\right)/\rho_{N}$ close to the superconducting transition for the ${\mathrm{Cu}}_{0.20}$ sample in constant magnetic fields from 0–1.8*T*. (**c**) The composition *x* dependence of the upper critical fields at zero temperature $\mu_{0}H_{c2}\left(0\right)$ (see panel (**a**) for details). Inset: $\mu_{0}H_{c2}\left(0\right)$ vs. $T_{c}$ for all samples; the dashed line corresponds to the Pauli paramagnetic limit $\mu_{0}H_{c2}^{\mathrm{Pauli}}=1.83T_{c}$. (**d**–**f**) The temperature dependence of the upper critical fields $\mu_{0}H_{c2}\left(T\right)$ for ${\left(\mathrm{TiZrNbNi}\right)}_{1-x}{\mathrm{Cu}}_{x}$, ${\left(\mathrm{TiZrNbCu}\right)}_{1-x}{\mathrm{Co}}_{x}$ and three additional alloys with fixed (CuNi) content. The lines represent the initial slopes of the upper critical field ${\left(\mu_{0}dH_{c2}/dT\right)}_{T=T_{c}}$.

All our samples, except for ${\mathrm{Cu}}_{0.50}$ and ${\mathrm{Co}}_{0.43}$, were superconducting above 300 mK. Variation of the superconducting transition temperatures $T_{c}$, defined as the temperature at which the resistivity drops to half the normal resistivity right above the transitions $\rho \left(T_{c}\right)=0.5\rho_{N}$, with composition can be found in Figure 2a. The width of the resistive transitions $\Delta T_{c}$ varied between 0.4 K for ${\mathrm{Co}}_{0.20}$ and 0.04 K for ${\mathrm{Ti}}_{0.30}$, where $\Delta T_{c}$ is defined as the temperature interval between $0.1\rho_{N}$ and $0.9\rho_{N}$. As previously noted [21], the $T_{c}$s values are lower than one would expect for binary alloys of similar composition.

On the other hand, the monotonic decrease of $T_{c}$ with increasing TL content, with the exception of ${\mathrm{Cu}}_{0.0}$, is consistent with other amorphous [21,27,37,38,39] and crystalline [40,41] TE-TL alloys. The ${\mathrm{Cu}}_{0.0}$ sample was noted for containing small amounts of a nanocrystalline phase in the amorphous matrix [20], which could affect its results. Furthermore, a lower value of $T_{c}$ for ${\mathrm{Ti}}_{0.30}$ compared to ${\mathrm{Zr}}_{0.30}$ and ${\mathrm{Nb}}_{0.30}$ is consistent with measurements on ${\left({\mathrm{Zr}}_{1-x}{\mathrm{Ti}}_{x}\right)}_{0.78}{\mathrm{Ni}}_{0.22}$ and ${\left({\mathrm{Zr}}_{1-x}{\mathrm{Nb}}_{x}\right)}_{0.78}{\mathrm{Ni}}_{0.22}$ [39], which showed a significant decrease of $T_{c}$ with increasing Ti content, while increasing the Nb content first increased and then decreased $T_{c}$ values.

The above-mentioned analogous behaviour of $T_{c}$ with TL content in both our and the binary amorphous TE-TL alloys strongly suggests that the same underlying mechanism is responsible for the suppression of $T_{c}$ in these systems. It has been argued that the change of $T_{c}$ with composition in TE-TL is primarily due to changes in the split-band structure of the density of states, which is seen in the UPS spectra [19,21,39]. However, a full understanding of $T_{c}$ dependence on alloy content is still lacking, and further research is needed on a wider range of different amorphous systems.

In Figure 2b, we present the temperature dependence of the normalized resistivity $\rho \left(T\right)/\rho_{N}$ in the vicinity of the superconducting transition for a ${\mathrm{Cu}}_{0.20}$ sample in a magnetic field. With an increasing magnetic field, the transition temperature $T_{c}$ drops, and the transition width increases, with the broadening becoming stronger as the temperature decreases. Therefore, to avoid the effects of this broadening, when determining the transition temperatures at different magnetic fields $T_{c}\left(\mu_{0}H\right)$, we opted to use a different resistivity criteria $\rho \left(T_{c}\left(\mu_{0}H\right)\right)=0.9\rho_{N}$. From these measurements, we then determined the temperature dependence of the upper critical fields $\mu_{0}H_{c2}\left(T\right)$, which are shown in Figure 2d–f. The lines on these figures represent the initial slopes of the upper critical field ${\left(\mu_{0}dH_{c2}/dT\right)}_{T=T_{c}}$.

The absolute values of the initial slopes decrease for ${\mathrm{Cu}}_{x}$ samples from 2.6 TK^−1^ to 2.3 TK^−1^ and increase for ${\mathrm{Co}}_{x}$ samples from 2.7 TK^−1^ to 3.2 TK^−1^ (except for the ${\mathrm{Co}}_{0.20}$ sample, which had a value around 2.5 TK^−1^). The ${\mathrm{Ti}}_{0.30}$ slope at 2.7 TK^−1^ was somewhat higher than that of ${\mathrm{Zr}}_{0.30}$ at 2.5 TK^−1^ and ${\mathrm{Nb}}_{0.30}$ at 2.4 TK^−1^.

From these slopes, we can estimate the upper critical fields at zero temperature using the Werthamer–Helfand–Hohenberg (WHH) approximation in the dirty limit [42]:

(1) $$\mu_{0}H_{c2}\left(0\right)=-0.69T_{c}\;{\left(\mu_{0}\frac{dH_{c2}}{dT}\right)}_{T=T_{c}}\;,$$

as shown in Figure 2c. The $\mu_{0}H_{c2}\left(0\right)$ values for all samples are close to the Pauli paramagnetic limit $\mu_{0}H_{c2}^{\mathrm{Pauli}}=1.83T_{c}$ (see inset of Figure 2c), which indicates that superconductivity is destroyed by paramagnetic pair breaking when the Zeeman energy becomes comparable to the superconducting gap energy.

### 3.3. Temperature Dependence of Resistivity

A small negative temperature coefficient of resistivity for strong scattering amorphous alloys (≥150 μΩ cm) is a long-standing issue; for a detailed discussion, see, for example, [32]. For amorphous alloys at temperatures ≳30 K, it is argued [25,26,32,43] that the temperature dependence of conductivity primarily arises from weak localization—specifically from the temperature variation of the inelastic scattering time $\tau_{i}$. When electron–phonon interactions are the dominant contribution to $\tau_{i}$—a reasonable assumption for amorphous metals—two temperature dependence regimes appear: $\sigma ∼\sqrt{T}$ for $T>\Theta_{D}$ and σ∼T for $T<\Theta_{D}$. We previously analysed the resistivity data for ${\mathrm{Ni}}_{x}$ alloys and found reasonably good agreement with these temperature variations above and below 100 K [21].

While the resistivity of our alloys presented here shows similar temperature behaviour, we suggest another origin for the observed ρ(T) temperature dependence: we argue that the total resistivity can be described by two parallel conductance channels in the form of:

(2) $$1/\rho \left(T\right)=1/\rho_{\mathrm{Metal}}\left(T\right)+1/\rho_{\mathrm{VRH}}\left(T\right)\;.$$

**Figure 3:**
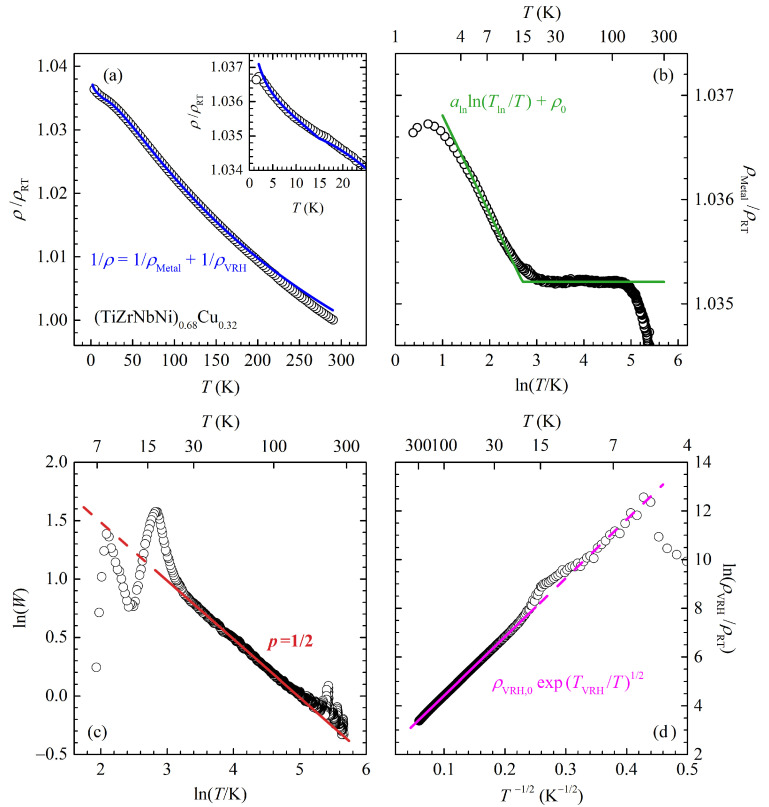
The results of fitting the measured temperature dependence of the ${\mathrm{Cu}}_{0.32}$ alloy resistivity to the model with two parallel conductance channels. The points are measured data, and the lines are fits. See the text for details. (**a**) The temperature dependence of the normalized resistivity $\rho /\rho_{\mathrm{RT}}$. Inset: low-temperature detail of the main panel. (**b**) The temperature dependence of the metallic part $\rho_{\mathrm{Metal}}\left(T\right)/\rho_{\mathrm{RT}}$. (**c**) The ‘special logarithmic derivative’ plot of the VRH-like contribution of the resistivity (see Equation (4)) with a p=1/2 slope. (**d**) Plot of the VRH-like contribution $\ln(\rho_{\mathrm{VRH}}/\rho_{\mathrm{RT}})$ vs. $1/\sqrt{T}$.

The $\rho_{\mathrm{Metal}}\left(T\right)$ is expected to be an ordinary metallic-like contribution. As our systems are disordered ones, we expect it to have a nearly constant value $\rho_{0}$ at higher temperatures. The second term, $\rho_{\mathrm{VRH}}\left(T\right)$, is another contribution, which we argue is of the variable range hopping origin. Figure 3 shows the result of fitting our ρ(T) data for the ${\mathrm{Cu}}_{0.32}$ alloy to Equation (2). (From here on: points denote measured/calculated data, and lines are fitting curves.) The top left panel (a) shows the overall raw measured data with the fitting curve. The top right panel (b) shows the normalized metallic contribution $\rho_{\mathrm{Metal}}\left(T\right)/\rho_{\mathrm{RT}}$ calculated with the fitting procedure. As expected, in the high temperature range, from around 20 to 200 K, we have a temperature-independent contribution.

Although it seems that this behaviour breaks at temperatures above 200 K, we propose that this is likely a consequence of very small variations of the overall resistivity in this temperature range, where a small error in the measured data can have a seemingly large effect (for example, the influence of extrinsic effects on resistance data, such as contact deterioration and thermal expansion of the sample, become significant compared to the VRH-like contribution, which, at room temperature, changes the total resistivity by only 3–5% from the constant $\rho_{0}$ value). Therefore, we argue that a temperature-independent $\rho_{\mathrm{Metal}}\left(T\right)$ should extend above T≳200 K.

After subtraction of the $\rho_{\mathrm{Metal}}\left(T\right)$ from the measured ρ(T), we are left with $\rho_{\mathrm{VRH}}\left(T\right)$, which is shown on the bottom right panel (d) of Figure 3 as the temperature dependence of $\rho_{\mathrm{VRH}}\left(T\right)/\rho_{\mathrm{RT}}$. It is worth noting that the VRH contribution to the overall ρ(T) is rather small, as its values are 20–30 times larger than $\rho_{\mathrm{Metal}}\left(T\right)$ at room temperature. From the plot, it is clear that $\rho_{\mathrm{VRH}}\left(T\right)$ can be described by a VRH-like temperature dependence

(3) $$\rho_{\mathrm{VRH}}\left(T\right)=\rho_{\mathrm{VRH},0}\;\exp{\left(\frac{T_{\mathrm{VRH}}}{T}\right)}^{p}\;,$$

with exponent p=1/2, for T≳20 K. (The $T_{\mathrm{VRH}}$ and $\rho_{\mathrm{VRH},0}$ are fitting parameters). Although here, we have set p=1/2 by hand, this exponent can be extracted independently using a so-called ‘special logarithmic derivative’ (see, for example, [44,45]), where one calculates:

(4) $$W=-\frac{d(\ln\rho_{\mathrm{VRH}})}{d(\ln T)}=p{\left(\frac{T_{\mathrm{VRH}}}{T}\right)}^{p}\;,$$

and then the slope of the ln(W)−ln(T) plot gives the value of *p*. This is shown on the bottom left panel (c) of Figure 3, where we again unequivocally obtain p=1/2 for T≳20 K. Here, we emphasize that the above fitting procedure is unique in a sense that only one choice of the $\rho_{\mathrm{Metal}}\left(T\right)$ values give VRH behaviour for $\rho_{\mathrm{VRH}}\left(T\right)$ with unique p=1/2.

**Figure 4:**
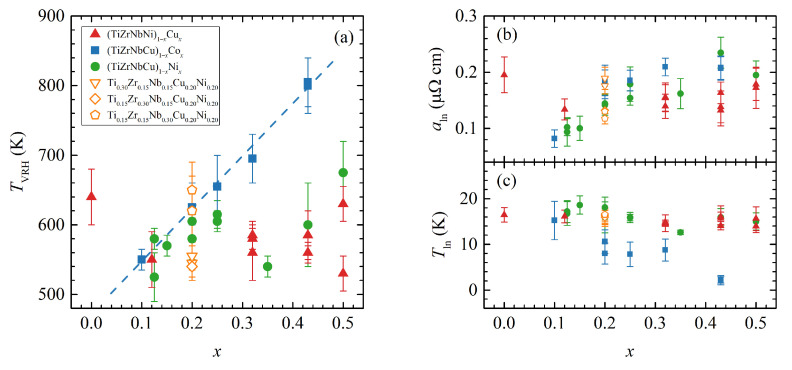
(**a**–**c**) The composition *x* dependence of the fitting parameters $T_{\mathrm{VRH}}$, $a_{\ln}$ and $T_{\ln}$, respectively, for ${\left(\mathrm{TiZrNbNi}\right)}_{1-x}{\mathrm{Cu}}_{x}$ (red triangles), ${\left(\mathrm{TiZrNbCu}\right)}_{1-x}{\mathrm{Co}}_{x}$ (blue squares), ${\left(\mathrm{TiZrNbCu}\right)}_{1-x}{\mathrm{Ni}}_{x}$ (green circles) and three additional alloys with fixed (CuNi) content (open triangles, diamonds and pentagons). The dashed line is a guide to the eye. Multiple points for the same *x* correspond to multiple measured samples.

The described fitting procedure can be satisfactorily applied to all our alloys, ${\mathrm{Cu}}_{x}$, ${\mathrm{Co}}_{x}$ and alloys with a fixed (CuNi) content, resulting in consistent values of fitting parameters. In addition, we reanalysed the resistivity data for ${\mathrm{Ni}}_{x}$ as previously reported in [21]. Figure 4 shows the fitting parameters $\rho_{\mathrm{VRH}}\left(T\right)$ (a), $a_{\ln}$ (b) and $T_{\ln}$ panel (c) dependence on the studied alloys (concentration). $T_{\mathrm{VRH}}$ is virtually constant for all alloys, except for ${\mathrm{Co}}_{x}$, which suggests a linear increase with Co concentration. $a_{\ln}$ is around 0.15 μΩ cm, and $T_{\ln}$ is around 15 K; in our opinion, this indicates that the entire idea of two parallel conducting channels, one being ‘metallic’ and other VRH-like, might be physically sound.

From the physical standpoint, the metallic contribution of the conductivity is clear. However, the origin of this VRH-like conduction mode and particularly the p=1/2 exponent is not immediately apparent. In a rather simple picture, localized states formed due to Anderson localization (driven by chemical and structural disorder), form randomly distributed insulating regions throughout the sample. These insulating regions then contribute to the VRH conductivity channel in the observed temperature dependence of resistivity.

It is well-known that the Anderson localization can lead to either a Mott [46] or an Efros–Shklovskii VRH mechanism [47]. In Mott VRH, the exponent *p* is dependent on the underlying system dimensionality and, for 3D systems, should be p=1/4, while the value we found, p=1/2, is characteristic for 1D systems, which clearly is not the case (our alloys are 3D). On the other hand, the Efros–Shklovskii VRH formalism appears more promising as it predicts a p=1/2 exponent irrespective of the dimensionality of the system. However, the temperature range (>20 K) in which we have a VRH-like conduction mode is higher than temperatures where this mechanism is usually observed (on the order of 10 K or less) [47,48].

A VRH-like temperature dependence in resistivity and a p=1/2 exponent were also obtained in granular metals (composite materials of conductive metallic regions embedded in an insulating matrix). In these systems, the hopping occurs between isolated metallic regions, which results in single carrier charging of those regions and an associated Coulomb penalty that results in the p=1/2 exponent—similar to the Efros–Shklovskii formalism but on a larger distance scale (see [49,50] for experimental details and [48,51] for theoretical considerations).

**Figure 5:**
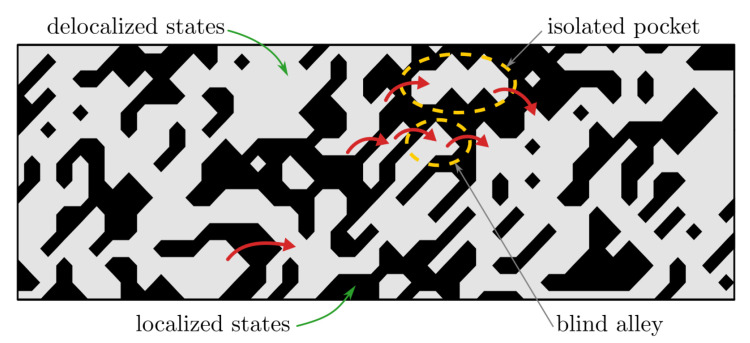
Illustration of the origin of the variable range hopping conductance contribution (see text for details). Gray area: the conductive region, corresponding to the delocalized states. Black area: nonconductive region due to the localization (driven by chemical and structural disorder). Red arrows: hopping between isolated pockets and/or blind alleys.

We propose that this is similar to our alloys: while delocalised states form an infinite cluster, the random spatial distribution of the aforementioned localised states (insulating regions) results in the appearance of finite clusters (isolated pockets) as well as dead ends (blind alleys) (see Figure 5), which do not contribute to the metallic conductivity, simultaneously increasing the resistivity [47]. Such isolated pockets and blind alleys could act as metallic regions in granular metals with localised states acting as the insulating regions. Hopping between these features could then result in a VRH-like conduction mode, parallel to the metallic conduction in the infinite cluster.

Furthermore, in the granular metal theory of VRH, $T_{\mathrm{VRH}}$ is dependent on the dielectric constant of the insulating region and, more importantly, on the (volume) ratio of the insulating and metallic regions [49]. This would indicate that the observed $T_{\mathrm{VRH}}$ increase with Co content in our ${\mathrm{Co}}_{x}$ alloys could be the result of an increase of the ratio of localised to delocalized states, which is, in turn, consistent with the increase of $\rho_{\mathrm{RT}}$ with Co content.

Finally, it is interesting to note increasing $\rho_{\mathrm{Metal}}\left(T\right)$ for temperatures below 20 K; see the top right panel (b) of Figure 3. One can argue that this temperature dependence is proportional to lnT, which is often found in metallic glass alloys. Several suggestions have been proposed to explain this resistivity behaviour in those systems, for example, the Kondo effect (∼ln*T*), scattering from two-level tunnelling states (∼ln*T* or ∼*T*) and electron–electron interaction effects (∼*T*) [32]. However, since the low-temperature resistivity (T≲20 K) of our samples could be equally well-fitted to a $\sqrt{T}$ dependence with a similar qualitative behaviour of corresponding fitting parameters, we feel that more work and results from other experimental techniques are required for a better understanding.

## 4. Conclusions

In this manuscript, the results of a systematic study of electrical resistivity, superconductive transitions and the Hall effect of amorphous samples of two quinary TE-TL alloy systems ${\left(\mathrm{TiZrNbNi}\right)}_{1-x}{\mathrm{Cu}}_{x}$ and ${\left(\mathrm{TiZrNbCu}\right)}_{1-x}{\mathrm{Co}}_{x}$ and three samples with the nominal compositions ${\mathrm{Ti}}_{0.30}{\mathrm{Zr}}_{0.15}{\mathrm{Nb}}_{0.15}{\mathrm{Cu}}_{0.2}{\mathrm{Ni}}_{0.2}$, ${\mathrm{Ti}}_{0.15}{\mathrm{Zr}}_{0.30}{\mathrm{Nb}}_{0.15}{\mathrm{Cu}}_{0.2}{\mathrm{Ni}}_{0.2}$ and ${\mathrm{Ti}}_{0.15}{\mathrm{Zr}}_{0.15}{\mathrm{Nb}}_{0.30}{\mathrm{Cu}}_{0.2}{\mathrm{Ni}}_{0.2}$ in the temperature range of 0.3–300 K and in magnetic fields up to ±16*T* are presented and compared to those of binary and multicomponent TE-TL alloys.

All measured samples showed a high resistivity at room temperature (140–240 μΩ cm) and a small negative temperature coefficient of resistivity—both of which are in agreement with binary TE-TL alloys. The dependence of room temperature resistivities $\rho_{\mathrm{RT}}$ on composition *x* is similar to those for corresponding binary alloys, except for ${\mathrm{Co}}_{x}$ alloys, where the resistivity increase with Co content is more significant than in binary ${\mathrm{Zr}}_{1-x}{\mathrm{Co}}_{x}$ alloys. The results of the Hall effect measurements are in good agreement with binary TE-TL amorphous alloys. The measured Hall coefficient $R_{H}$ is temperature-independent and positive (except for ${\mathrm{Cu}}_{0.43}$). With the change in composition *x*, $R_{H}$ is nearly constant for ${\mathrm{Cu}}_{x}$ alloys, while, for ${\mathrm{Co}}_{x}$ alloys, $R_{H}$ decreases with increasing Co content and changes sign around x(Co)=0.34.

These changes, or lack thereof, are consistent with the shift in position of the peaks associated with 3D states of Cu and Co in the UPS spectra. Furthermore, for alloys with fixed (CuNi) content, $R_{H}$ increases with additional Ti content and decreases with additional Zr or Nb content. The observed superconducting transition temperatures $T_{c}$ are surprisingly low ($T_{c}\leq 1.94$ K) and decrease further with increasing TL (Cu and Co) content—the latter being consistent with other amorphous and crystalline TE-TL alloys. The upper critical fields at zero temperature $\mu_{0}H_{c2}\left(0\right)$ appear to be Pauli limited.

As for the temperature dependence of the resistivity—in particular, its increase with decreasing temperature—we present a model with two parallel conductance channels in the form $1/\rho \left(T\right)=1/\rho_{\mathrm{Metal}}\left(T\right)+1/\rho_{\mathrm{VRH}}\left(T\right)$ and show that, for T≳20 K, our data can be nicely fitted with a constant $\rho_{\mathrm{Metal}}\left(T\right)$ and variable range hopping, such as contribution $\rho_{\mathrm{VRH}}\left(T\right)$ with exponent p=1/2. From a physical standpoint, the temperature-independent $\rho_{\mathrm{Metal}}\left(T\right)$ agrees with the notion that the system is strongly disordered.

For the origin of $\rho_{\mathrm{VRH}}\left(T\right)$, we propose the idea that a portion of states near the Fermi level are localized due to the disorder, resulting in insulating and conductive metallic regions. The random spatial distribution of these localised states (insulating regions) throughout the samples creates isolated pockets and blind alleys, which do not contribute to the metallic conductivity but contribute to conductivity via a hopping mechanism akin to VRH in granular metals.

To conclude, we propose that measurements on amorphous thin films of varying thickness of these (Ti-Zr-Nb-Ni-Cu and Ti-Zr-Nb-Co-Ni) and similar alloys could provide further insight into the nature of the possible VRH regime that we observed in our alloys.

## Data Availability

The data presented in the figures are available on request from the M.K. and M.B.
